# Anti-TNF-*α* Therapy Suppresses Proinflammatory Activities of Mucosal Neutrophils in Inflammatory Bowel Disease

**DOI:** 10.1155/2018/3021863

**Published:** 2018-11-22

**Authors:** Cui Zhang, Weigang Shu, Guangxi Zhou, Jian Lin, Feifei Chu, Huili Wu, Zhanju Liu

**Affiliations:** ^1^Department of Gastroenterology, The Shanghai Tenth People's Hospital, Tongji University, Shanghai 200072, China; ^2^Department of Gastroenterology, Zhengzhou Central Hospital Affiliated to Zhengzhou University, Zhengzhou, Henan Province 450007, China

## Abstract

Neutrophils have been found to play an important role in the pathogenesis of inflammatory bowel disease (IBD), and anti-TNF-*α* mAb (i.e., infliximab) therapy is demonstrated to be effective in the induction of clinical remission and mucosal healing in these patients. However, how anti-TNF-*α* mAb regulates the functions of neutrophils is still unknown. Herein, we found that anti-TNF-*α* therapy significantly downregulated infiltration of neutrophils in inflamed mucosa of IBD patients. Importantly, anti-TNF-*α* mAb could inhibit neutrophils to produce proinflammatory mediators, such as ROS, calprotectin, IL-8, IL-6, and TNF-*α*. These data indicate that TNF-*α* plays a critical role in the induction of mucosal inflammatory response, and that blockade of TNF-*α* modulates intestinal homeostasis through balancing immune responses of neutrophils.

## 1. Introduction

Inflammatory bowel diseases (IBD), including ulcerative colitis (UC) and Crohn's disease (CD), are chronic relapsing inflammatory disorders affecting the gastrointestinal tract. The incidence of IBD in China keeps increasing in recent decades [1]. Although the pathogenesis of IBD is still elusive, it is generally considered that IBD is caused by inappropriate mucosal immune responses to commensal microbiota in genetically susceptible individuals [2–4]. Current medical therapies for IBD consist of 5-aminosalicylic acid (5-ASA), corticosteroids, immunosuppressants (e.g., azathioprine, cyclosporine, tacrolimus, and methotrexate), and biological agents. In the past decades, increasing numbers of biological monoclonal antibodies (mAbs) are emerging (e.g., infliximab, adalimumab, certolizumab pegol, vedolizumab, natalizumab, and ustekinumab) which have been proven to be an evolutionary approach in the management of human IBD [5–8].

As one of the anti-TNF-*α* mAbs, infliximab (IFX, Remicade®) is the earliest commercialized biological molecular agent for the treatment of IBD. It is a chimeric mouse-human monoclonal antibody, which is effective for blocking both soluble and transmembrane TNF-*α* (tmTNF-*α*). Previous studies have demonstrated that IFX can induce and maintain remission in 45%–70% of IBD patients who previously failed to steroids or immunosuppressants. Patients with recurrent IBD can maintain long-term mucosal healing with IFX and avoid or postpone surgery. By the neutralization of TNF-*α*, IFX has been found to be attributed to several biological effects in the treatment of IBD patients, such as induction of proinflammatory T helper (Th) cell apoptosis, inhibition of growth factor, and antibody production and regulation of adhesion molecular expression. Moreover, IFX can also promote regulatory IL-10-producing macrophages, which can further induce regulatory Th cell differentiation [9]. However, it is still unknown whether IFX can impact on any other innate immune cells (e.g., neutrophils) in IBD.

Neutrophils play a beneficial and detrimental role in intestinal inflammation [10]. On one hand, the functions of neutrophils include engulfing invasive pathogens, releasing antimicrobial peptides (AMPs), producing reactive oxygen species (ROS) and neutrophil extracellular traps (NET), and attacking invasive pathogens through phagocytosis and degranulation. Neutrophils can protect intestinal mucosa from infection in this manner. On the other hand, extravasation of neutrophils from peripheral blood to inflamed mucosa is a hallmark of IBD activity [11]. Without appropriately regulating early inflammatory response, neutrophils trigger an uncontrolled positive feedback amplification loop leading to tissue damage and resolution delay. Moreover, neutrophils also produce multiple proinflammatory cytokines (e.g., IL-1*β*, IL-6, TNF-*α*, and IFN-*γ*) and chemokines (e.g., IL-8, CCL3, and CCL4), which further recruit neutrophils and monocytes/macrophages to the inflamed sites [12]. Our recent work also demonstrated that CD177^+^ neutrophils are one of the main sources of IL-22 in the intestine, which is of importance in guaranteeing epithelial barrier integrity [13]. The regulatory mechanisms of IFX in regulating macrophage polarization and T cell differentiation in IBD have been elucidated carefully, but how it modulates neutrophils is still unknown.

In this study, we investigated the migration of neutrophils in IBD patients before and after treatment with anti-TNF-*α* mAb, and explored the regulatory role of anti-TNF-*α* in the production of proinflammatory mediators. Our results demonstrated that anti-TNF-*α* therapy significantly downregulated the infiltration of neutrophils in inflamed mucosa in CD patients who responded to IFX. Importantly, anti-TNF-*α* mAb markedly suppressed production of proinflammatory mediators, such as MPO, calprotectin, IL-8, IL-6, and TNF-*α*. Moreover, blocking TNF-*α* could remarkably induce neutrophil apoptosis in CD patients. These data indicate that anti-TNF-*α* therapy ameliorates mucosal inflammation of IBD patients by downregulating activation and migration of neutrophils in gut mucosa.

## 2. Materials and Methods

### 2.1. Subjects and Sample Collection

All peripheral blood and intestinal tissue samples were collected from IBD patients and healthy donors in the Department of Gastroenterology, the Shanghai Tenth People's Hospital of Tongji University (Shanghai, China) from May 2016 to March 2018. EDTA anticoagulated blood samples (10–15 ml) were harvested from patients with active CD (A-CD, *n* = 26), patients with CD in remission (R-CD, *n* = 22), patients with active UC (A-UC, *n* = 28), patients with UC in remission (R-UC, *n* = 16), and healthy volunteers (HC, *n* = 24) after overnight fasting. The diagnosis of CD or UC was based on clinical characteristics, radiological examination, endoscopy, and histological findings. The clinical characteristics of these patients with IBD are shown in Table 1.

This work was approved by the Institutional Review Board for Clinical Research of the Shanghai Tenth People's Hospital of Tongji University. Written informed consent was also obtained from all subjects before the study.

### 2.2. Anti-TNF-*α* mAb Therapy in Patients with Active CD

22 patients with active CD were recruited and treated with anti-TNF-*α* mAb (IFX; Cilag AG, Schaffhausen, Switzerland) at weeks 0, 2, and 6. Peripheral blood samples and paired intestinal mucosal biopsies were collected before and 12 weeks after the first IFX administration. According to the criteria of CDAI, these patients were classified into 2 groups, including the response group (15 patients (68.2%) achieved clinical response to IFX) and the failure group (7 patients (31.8%) failed to respond to IFX). Active CD was defined as CDAI > 150. Clinical remission was defined as CDAI < 150, and clinical response was defined as CDAI decreased by at least 70 points at the evaluation time point compared to the baseline index.

### 2.3. Immunohistochemistry

Fresh intestinal biopsies from IBD patients and healthy donors were fixated with 10% paraformaldehyde for 24 h and embedded with paraffins followed by slicing to 5 *μ*m-thick sections. After deparaffiniztation and rehydration, antigen retrieval was performed with pH = 6 sodium citrate buffer or pH = 9 Tris-EDTA buffer. Endogenous peroxidase was inhibited by 3% H_2_O_2_. After blocking with 10% normal goat serum for 30 min at room temperature, these sections were incubated with rabbit anti-human CD66b mAb (Abcam, dilution 1 : 200), rabbit anti-human MPO (Abcam, 1 : 1000), or control IgG at 4°C overnight. After rinsing in 0.03% Triton-PBS buffer, the sections were incubated with HRP-conjugated anti-rabbit/mouse IgG (Dako) at room temperature for 40 min. After staining with diaminobenzidine (DAB), the sections were counterstained with haematoxylin.

### 2.4. Isolation of Neutrophils, Antibodies, and Flow Cytometry

Peripheral neutrophils from patients with IBD were isolated with Ficoll-Hypaque (GE Healthcare, Piscataway, NJ, USA) density gradient centrifugation as described previously [11]. Briefly, peripheral blood was collected in EDTA-anticoagulated tubes and slowly laid on the surface of Ficoll, followed by gradient centrifuged at 2000 rpm at 20°C. The lowest layer was collected and neutrophils were obtained after incubating with red blood cell lysis buffer (BD Biosciences, San Diego, CA, USA). Cells were incubated with Fc block antibody (BioLegend, San Diego, CA, USA) in FACS buffer for 10 min to block nonspecific binding, followed by staining with respective cell surface antibodies at 4°C for 30 min. Primary antibodies used in this study included PE-anti-CD66b (BioLegend) and APC-CY7-anti-LiveDead (Life Invitrogen, MA, USA). Data were acquired on a BD FACSCanto II and further analyzed with FlowJo 10.0. Apoptosis analysis was performed as follows: Neutrophils were collected and stimulated with indicated stimuli. Cells were stained with PI (BioLegend) and APC-Annexin V (BioLegend) for 15 min at room temperature. Data were then acquired on BD FACSCanto II.

### 2.5. Quantitative Real-Time PCR

Total RNA of neutrophils was extracted with TRIzol. The concentration and purity of RNA were determined by a NanoVue spectrophotometer (GE Healthcare). The cDNA was synthesized with a 5x All-In-One RT MasterMix Kit (abm) based on the manufacturer's instructions. PCR reaction conditions were manipulated as follows: 25°C for 10 min, 42°C for 15 min, and 85°C for 5 min. qRT-PCR was performed according to the SYBR green (TaKaRa) instructions and the reaction was performed by BD 7900. RT-PCR reaction conditions were as follows: 95°C for 1 min, 95°C for 15 s, and 60°C for 30 s, repeated for 40 cycles. All primers were synthesized by Huada BioTech (Shanghai, China) and GAPDH was used as housekeeping gene. qRT-PCR analysis was calculated with the 2^−ΔΔCt^ method.

### 2.6. Enzyme-Linked Immunosorbent Assay

Protein levels of cytokines and chemokines were measured by ELISA according to the manufacturer's instruction (BioLegend). Briefly, captured antibodies were incubated in 96-well plates at 4°C overnight. Nonspecific antigens were blocked with assay diluents. The standard and samples were added and incubated at 37°C for 2 h. After thoroughly washing with 0.05% Tween-PBS, the plates were incubated with detection antibodies for 1 h and HRP for 30 min. Finally, the color was developed with TMB and the value of OD was detected with a microplate spectrophotometer (BioTek).

### 2.7. In Vitro Culture of Neutrophils and Cytokine Analysis

5 × 10^6^ peripheral neutrophils were cultured *in vitro* in 1 ml RPMI 1640-10% FBS and LPS (200 ng/ml) was added to stimulate the cells in the presence of IFX (50 *μ*g/ml) or control human IgG (HIg 50 *μ*g/ml) at 37°C in 5% CO_2_ humidified air for 3 h. Cells were then collected for qRT-PCR. For the detection of protein levels of cytokines produced by neutrophils, 2 × 10^6^ neutrophils were stimulated with LPS (200 ng/ml) in the presence of IFX (50 *μ*g/ml) or control human IgG (HIg 50 *μ*g/ml) for 3 h. The culture medium was then replenished with 1 ml fresh RPMI 1640-10% FBS and cultured for another 24 h. Supernatants were collected for ELISA. For the detection of ROS and MPO produced by neutrophils, 2 × 10^6^ neutrophils were incubated in the presence of human IgG (HIg 50 *μ*g/ml) or IFX (50 *μ*g/ml) for 3 h. 1 × 10^4^ cells then were collected and incubated in HBSS in the presence of peroxidase and Amplex^@^ Red reagent in 96-well plates according to the manufacturer's instruction illustrated in the Amplex Red Hydrogen Peroxide/Peroxidase Assay Kit (Life Invitrogen).

### 2.8. Mucosal Biopsy Culture In Vitro

Fresh colon biopsies were obtained from patients with A-CD (*n* = 13) and A-UC (*n* = 13) during endoscopy and cultured ex vivo in 1 ml RPMI 1640-10% FBS medium in the presence of IFX or HIg (both at 50 *μ*g/ml) at 37°C in 5% CO_2_ humidified air for 24 h. Tissues were then collected for qRT-PCR.

### 2.9. Statistical Analysis

All data analyses were carried out by GraphPad Prism 5. Data were expressed as mean ± SEM, and at least 3 independent experiments were performed. Two-way ANOVA followed by Bonferroni's correction and paired or unpaired two-tailed Student's *t*-test was used for statistical comparisons. Statistical significance was defined as follows: ^∗^ *P* < 0.05, ^∗∗^ *P* < 0.01, and ^∗∗∗^ *P* < 0.001.

## 3. Results

### 3.1. IFX Therapy Markedly Downregulates Activities of Neutrophils in Peripheral Blood and Inflamed Mucosa in Patients with IBD

**Figure 1:**
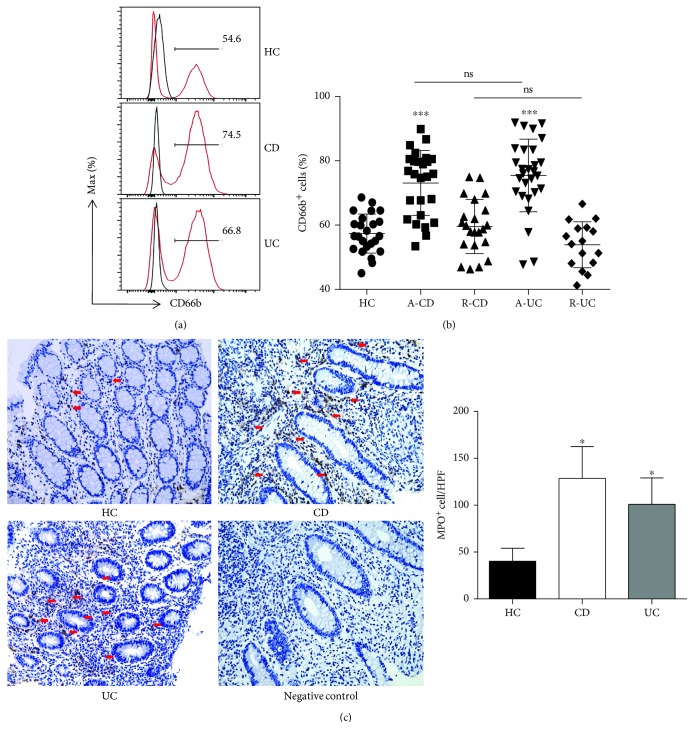
Neutrophils are more activated in the peripheral blood and intestinal mucosa of active IBD patients. (a, b) Peripheral blood samples were collected from healthy donors (HC, *n* = 24), patients with active CD (A-CD, *n* = 26), patients with CD in remission (R-CD, *n* = 22), patients with active UC (A-UC, *n* = 28), and patients with UC in remission (R-UC, *n* = 16). The whole blood excluding RBC was analyzed by flow cytometry. ^∗∗∗^ *P* < 0.001 compared with HC. (c) Immunohistochemical staining of MPO in inflamed intestinal mucosa from healthy control (HC, *n* = 10), patients with A-CD (*n* = 10), and patients with A-UC (*n* = 10). The arrows indicate MPO^+^ cells. Original magnification × 200. ^∗^ *P* < 0.05 compared with HC.

Neutrophils have been found to be significantly activated in peripheral blood and inflamed mucosa in active IBD patients. We first analyzed the percentage of CD66b^+^ neutrophils in peripheral blood and found that it was significantly increased in patients with active IBD compared with that in patients with IBD in remission and healthy donors (Figures 1(a) and 1(b)). Consistently, immunohistochemical staining further revealed that the infiltration of neutrophils was also significantly increased in the lamina propria of inflamed intestinal mucosa from patients with active IBD compared with healthy controls (Figure 1(c)).

**Figure 2:**
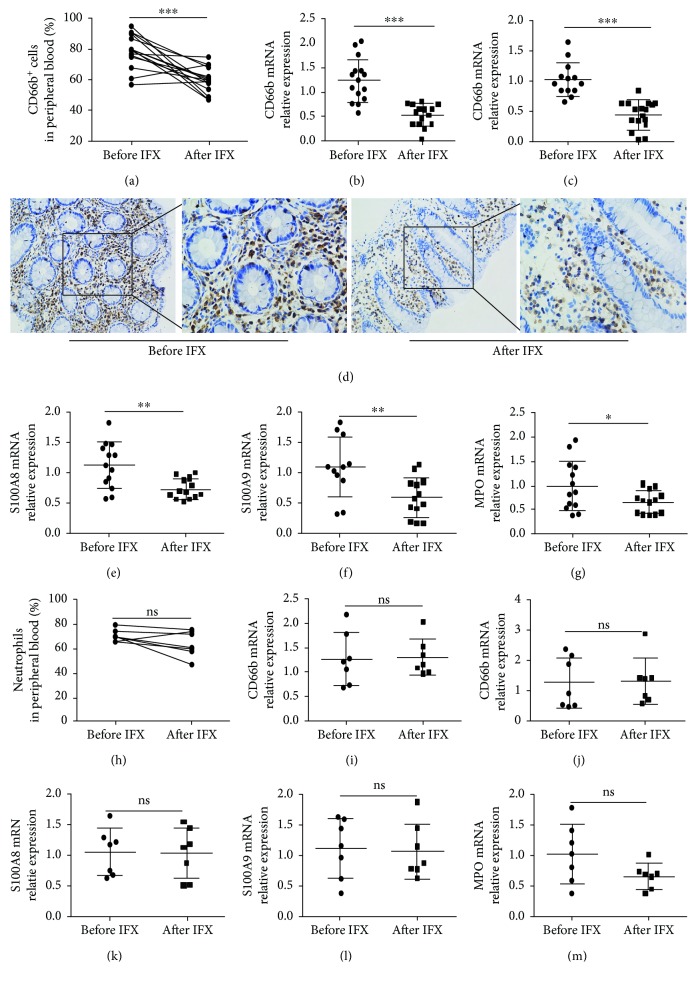
The activity of neutrophils is inhibited in the peripheral blood, and the infiltration of neutrophils is decreased in the intestinal mucosa of CD patients after anti-TNF-*α* treatment. Response group: (a) percentages of CD66b^+^ neutrophils in the peripheral blood of patients with active CD (*n* = 15) before and after IFX treatment; (b) expression of CD66b mRNA in the peripheral blood neutrophils from patients with active CD (*n* = 15) before and after IFX treatment by qRT-PCR; (c) expression of CD66b mRNA in intestinal mucosa from patients with active CD (*n* = 13) before and after IFX treatment by qRT-PCR; (d) immunohistochemical staining of CD66b in the intestinal mucosa of active CD (*n* = 10) before and after IFX treatment; and (e) expression of S100A8, (f) S100A9, and (g) MPO in the intestinal mucosa of patients with active CD (*n* = 13) before and after IFX treatment. Failure group: percentage (h) and expression of CD66b (i) of peripheral blood neutrophils from active CD patients (*n* = 7) before and after IFX. Expression of CD66b (j), S100A8 (k), S100A9 (l), and MPO (m) in intestinal mucosa was detected by qRT-PCR from CD patients in the failure group. ^∗^ *P* < 0.05, ^∗∗^ *P* < 0.01, and ^∗∗∗^ *P* < 0.001.

Next, we investigated whether neutrophils could be modulated after receiving anti-TNF-*α* mAb (IFX) in patients with CD. Interestingly, we found that the percentage of CD66b^+^ neutrophils and expression of CD66b were significantly decreased in peripheral blood and inflamed mucosa of patients with active CD at the 12th week after anti-TNF-*α* therapy in the response group (Figures 2(a)–2(d)), while they did not change in the failure group (Figures 2(h)–2(j)). Moreover, we also found that the anti-TNF-*α* treatment markedly downregulated the expression of calprotectin (e.g., S100A9 and S100A8) and MPO in inflamed mucosa in the response group (Figures 2(e)–2(g)). On the contrary, they did not show any significant difference in inflamed mucosa in the failure group (Figures 2(k)–2(m)).

**Figure 3:**
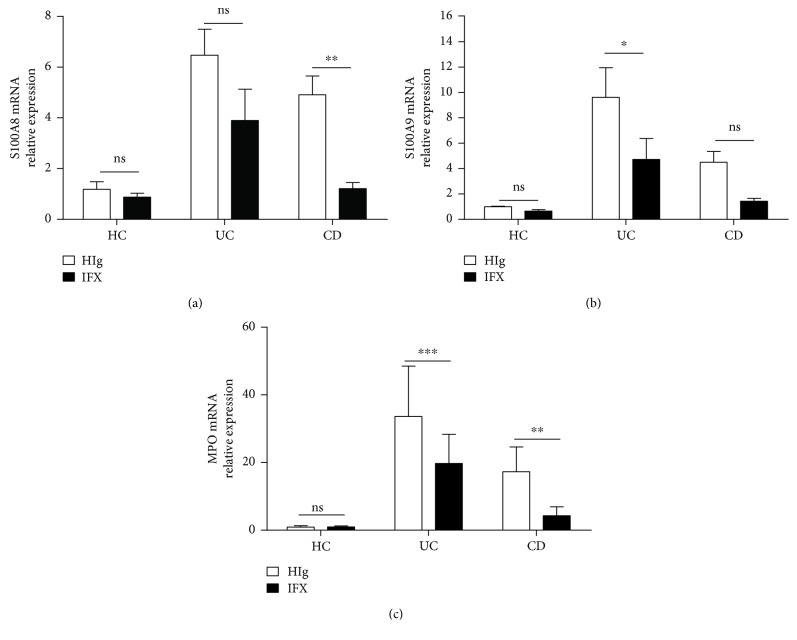
IFX suppresses the expression of neutrophil-derived S100A8, S100A9, and MPO in intestinal mucosa. Fresh colon biopsies were collected from inflamed mucosa of patients with active CD (*n* = 12) and active UC (*n* = 12), and cultured ex vivo with IFX or control human IgG (HIg) (both at 50 *μ*g/ml) for 24 h. Tissues were harvested for detection of expression of S100A8 (a), S100A9 (b), and MPO (c) by qRT-PCR and compared with healthy controls (*n* = 5). ^∗^ *P* < 0.05 and ^∗∗^ *P* < 0.01.

Additionally, we also collected fresh inflamed intestinal mucosal biopsies from patients with active IBD during endoscopy and cultured *in vitro* in the presence of IFX or HIg for 24 h. Intriguingly, we found that anti-TNF-*α* treatment markedly decreased expression of neutrophil-derived MPO and calprotectin in inflamed mucosa from patients with IBD (Figures 3(a)–3(c)).

Collectively, these data indicate that anti-TNF-*α* mAb therapy markedly suppresses activities and infiltration of neutrophils in peripheral blood and inflamed mucosa in IBD patients. Blockade of TNF-*α* reduces the release of proinflammatory mediators both *in vivo* and ex vivo.

### 3.2. Anti-TNF-*α* Therapy Inhibits Neutrophils to Produce Proinflammatory Mediators in IBD Patients

**Figure 4:**
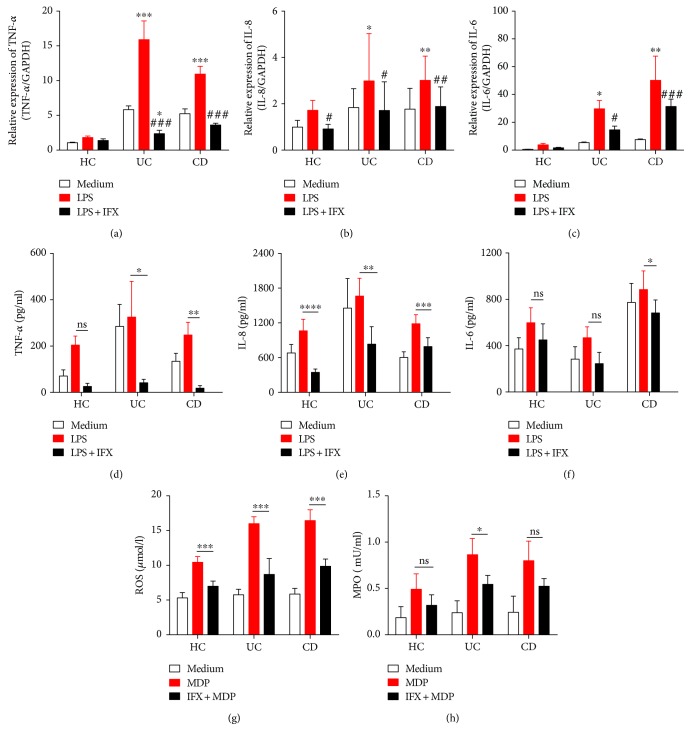
Anti-TNF-*α* therapy inhibits peripheral neutrophils to produce proinflammatory cytokines, chemokines, ROS, and MPO. Peripheral neutrophils (5 × 10^6^) from healthy donors (*n* = 10), patients with active CD (*n* = 10), and active UC (*n* = 10) were stimulated by LPS (200 ng/ml) and incubated with or without IFX (50 *μ*g/ml) for 3 h. Cells were collected and expressions of TNF-*α* (a), IL-8 (b), and IL-6 (c) were detected by qRT-PCR. Peripheral neutrophils (2 × 10^6^) from healthy donors (*n* = 6), patients with active CD (*n* = 11), and patients with active UC (*n* = 4) were simulated with LPS (200 ng/ml) and incubated with or without IFX (50 *μ*g/ml) for 3 h. Culture media were replenished and incubated for another 24 h. Supernatants and protein production of TNF-*α* (d), IL-8 (e), and IL-6 (f) were measured by ELISA. Peripheral neutrophils (1 × 10^4^) isolated from healthy donors (*n* = 5), patients with active CD (*n* = 5), and patients with active UC (*n* = 5) were measured for ROS (h) and MPO (i) with Amplex Red Hydrogen Peroxide Assay Kit. ^∗^ *P* < 0.05, ^∗∗^ *P* < 0.01, and ^∗∗∗^ *P* < 0.001 compared with medium control and ^#^ *P* < 0.05, ^##^ *P* < 0.01, and ^###^ *P* < 0.001 compared with LPS stimulation.

Although we have already proved that IFX could downregulate production of proinflammatory mediators in inflamed intestinal mucosa from patients with IBD, whether IFX has an impact on neutrophils is still undetermined. To this end, we isolated peripheral neutrophils from IBD patients and measured the production of proinflammatory mediators. As shown in Figures 4(a)–4(f) and Supplementary Figures 2(a)–2(f), proinflammatory mediators (e.g., TNF-*α*, IL-8, and IL-6) were significantly suppressed by IFX treatment, while TGF-*β* and IL-17A were not altered. Moreover, we also observed that MPO and ROS production were remarkably downregulated under treatment with IFX (Figures 4(g) and 4(h)). Taken together, these data indicate that anti-TNF-*α* therapy indeed suppresses neutrophils to produce proinflammatory mediators.

### 3.3. Anti-TNF-*α* Therapy Suppresses Migration of Neutrophils in Patients with Active CD

**Figure 5:**
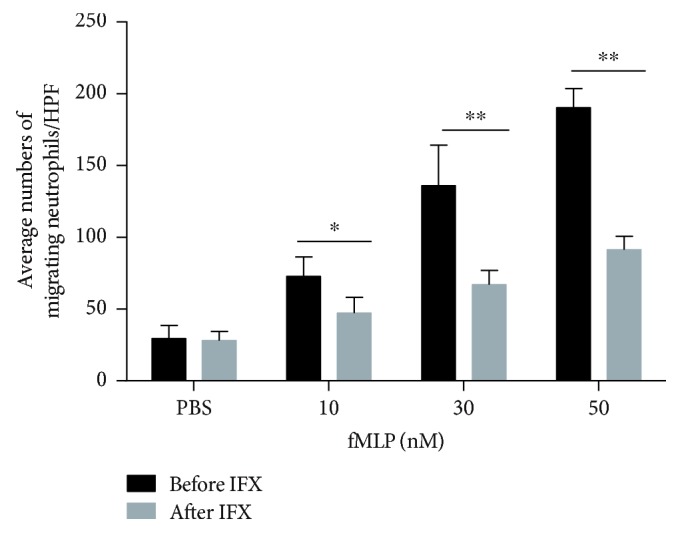
Anti-TNF-*α* therapy suppresses neutrophil migration in active CD patients. Peripheral neutrophils (5 × 10^5^) were isolated from patients with active CD (*n* = 4) and measured with an 8 *μ*m-Transwell plate under attraction with fMLP (10, 30, and 50 nM) for 30 min. The histogram represents the number of migrating neutrophils per high-power field (HPF). ^∗^ *P* < 0.05 and ^∗∗^ *P* < 0.01.

Since several lines of evidence have proved that large amounts of neutrophils infiltrated in inflamed mucosa during active inflammatory response in IBD [12], we sought to determine the effects of anti-TNF-*α* therapy on the migration of neutrophils. To this end, peripheral neutrophils were isolated and the capacity of migration was examined subsequently by a Transwell model. We found that anti-TNF-*α* treatment significantly suppressed the migration of neutrophils compared with controls (Figure 5). Therefore, our data indicate that anti-TNF-*α* therapy could suppress the migration of neutrophils in patients with active CD.

### 3.4. Anti-TNF-*α* Therapy Promotes Apoptosis of Neutrophils in Patients with Active CD

**Figure 6:**
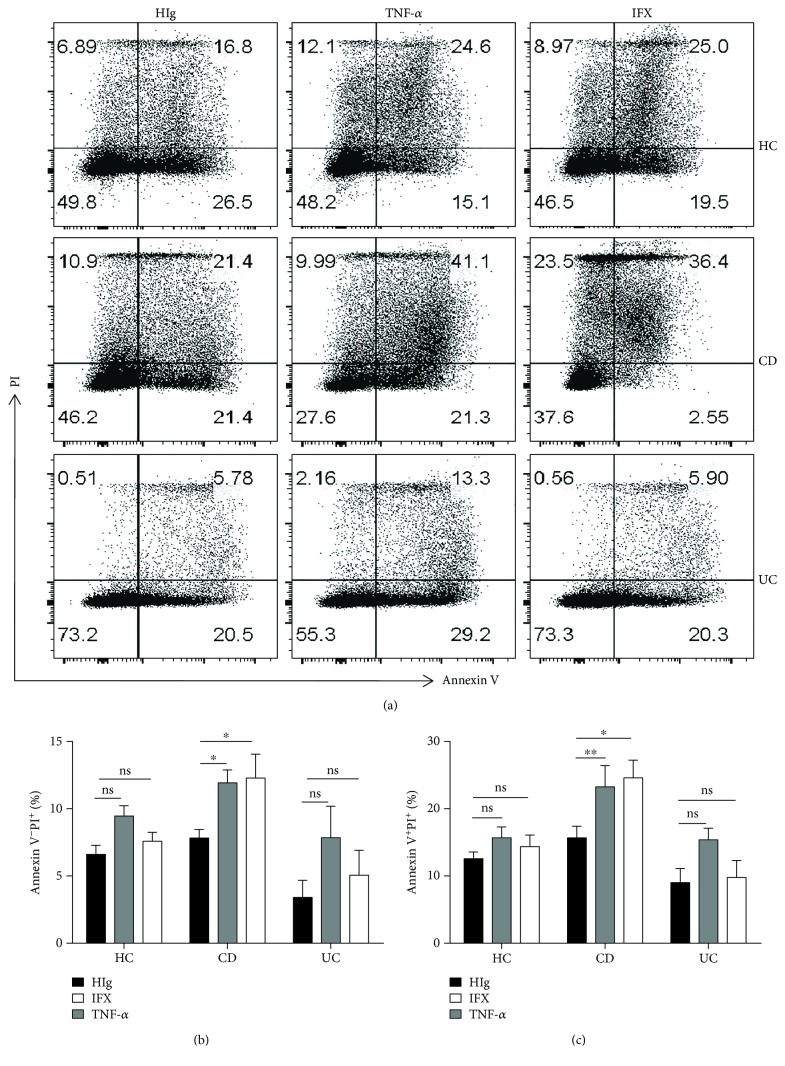
Anti-TNF-*α* therapy promotes apoptosis of neutrophils in active CD patients. (a–c) Peripheral neutrophils were isolated from healthy donors (*n* = 8), patients with active CD (*n* = 10), and patients with active UC (*n* = 5) and incubated with HIg (50 *μ*g/ml), IFX (50 *μ*g/ml), or TNF-*α* (20 ng/ml) for 24 h. Cells were collected and detected for apoptosis by flow cytometry. ^∗^ *P* < 0.05.

Our previous data have shown that the percentage of neutrophils was significantly decreased in peripheral blood from patients with active CD after IFX therapy. We hypothesized that IFX could not only inhibit neutrophils to migrate to an inflamed intestine but also promote apoptosis of peripheral neutrophils. Hence, we isolated peripheral neutrophils from patients with active IBD and assessed the apoptosis and necrosis of neutrophils. Interestingly, our results demonstrated that IFX could markedly promote apoptosis of neutrophils in patients with active CD compared with healthy donors and patients with active UC (Figures 6(a)–6(c)). Collectively, these data indicate that anti-TNF-*α* therapy promotes apoptosis of neutrophils in patients with CD.

## 4. Discussion

TNF-*α* is a critical proinflammatory cytokine in the pathogenesis of IBD and IFX is one of the anti-TNF-*α* mAbs, which is proved to be effective in IBD treatment [14]. IFX has been shown to markedly suppress activities of macrophages and T cells and restore epithelial barrier function [15]. However, its roles in regulating neutrophil function are still unclear. Our data demonstrated that IFX could significantly downregulate proinflammatory activities of neutrophils in either peripheral blood or inflamed intestinal mucosa in patients with active IBD.

Our previous studies have demonstrated that IFX treatment markedly regulates CD4^+^ T cell differentiation and induces intestinal mucosal CD4^+^ T cells to produce IL-22 which further maintains epithelial barrier integrity [16–18]. In addition, IFX can impede on adherence and chemotaxis of neutrophils [19, 20]. Consistent with previous reports, our results exhibited that infiltration and activities of neutrophils were markedly increased in peripheral blood and inflamed mucosa from patients with IBD. Given that 64% patients with moderate-to-severe CD who are refractory to steroids can achieve response to IFX therapy, our study demonstrated that 68.3% of the patients with active CD responded to IFX. Additionally, the activities of neutrophils were also observed to be restored in both peripheral blood and intestinal mucosa in the response group.

Neutrophil-derived inflammatory mediators play a decisive role in triggering inflammation. Neutrophils are a source of proinflammatory cytokines and chemokines such as IL-6, TNF-*α*, IFN-*γ*, and IL-8, although some cytokines are only produced under specific circumstances. In our study, we found that the production of those proinflammatory mediators could be efficiently inhibited by IFX. However, IFX did not impact on neutrophil's ability to produce TGF-*β*. Previous studies have reported that neutrophils do not secrete IL-6, unless they are stimulated with the ligand for TLR8 [21]. However, it is also mentioned that LPS can induce neutrophils to produce IL-6 at higher concentrations (≥100 ng/ml) [22]. Consistently, we found that neutrophils could produce detectable IL-6 when stimulated with LPS at a concentration of 200 ng/ml. Moreover, it has been reported that the TLR4/MyD88 signaling pathway is much upregulated in IBD patients and TLR4 gene polymorphisms are also associated with IBD susceptibility [23]. Thus, neutrophils from IBD patients are probably hyperreactive to LPS stimulation. Furthermore, neutrophils can produce a large quantity of noncytokine inflammatory mediators (e.g., matrix metalloproteases, MPO, ROS, calprotectin, and neutrophil elastase) [24]. Elevated S100A9 and S1008 are positively associated with the disease severity of IBD and persistent ROS production causes DNA damage and lipid peroxidation, which further result in intestinal epithelial disruption and impaired mucosal permeability [25, 26]. In addition, calprotectin and MPO are also produced by enterocytes and macrophages [27, 28]. To further illustrate that IFX negatively regulates neutrophils, we studied peripheral neutrophils and found that IFX indeed inhibited neutrophils to produce calprotectin, ROS, and MPO. Taken together, these results imply that IFX therapy can modulate the detrimental role of neutrophils in the pathogenesis of IBD.

Multistep processes have been shown to participate in the extravasation of neutrophils out of a vessel and migration to the inflamed site, including rolling, crawling, transendothelial migration, and chemotaxis [29]. Since the roles of neutrophils are dichotomous in the development of colitis, promoting the early migration of neutrophils into the crypts of the intestine ameliorates inflammation while the continuous influx of neutrophils will exacerbate it [30]. To elucidate the influence of IFX on the migration of neutrophils, we applied a Transwell model and found that IFX could inhibit the fMLP-induced migration of neutrophils. Both IL-8 and fMLP are critical chemotactic agents that are essential for neutrophils to migrate into inflamed mucosa [31]. As one of the GPCR agonists, fMLP can activate NF-*κ*B, MAPK, and PI3K/Akt signaling pathways, which play a critical role in the production of IL-8 in human neutrophils [32]. There is evidence to prove that blockade of tmTNF-*α* can significantly suppress production of IL-8, IL-6, and IL-1*β*, which can further downregulate NF-*κ*B and PI3K/Akt signaling pathways [33]. We hypothesized that IFX can simultaneously inhibit neutrophils to produce IL-8 and downregulate NF-*κ*B and PI3K/Akt signaling pathways, which contribute to decreased migration of neutrophils, leading to the resolution of inflammation in intestinal mucosa in CD patients. Therefore, more detailed mechanisms whereby IFX governs neutrophil migration need to be further investigated.

Neutrophils were once regarded as cells with a short lifespan in humans. After eliminating invading pathogens, neutrophils undergo appropriate apoptosis, which is a crucial process of inflammation resolution. Delayed neutrophil apoptosis is observed in patients with IBD, and proinflammatory mediators (e.g., G-CSF and IL-8) are associated with this phenomenon [34, 35]. It was once mentioned that IFX therapy can induce lamina propria mononuclear cell apoptosis [36, 37]. Interestingly, our experiments indicated that blockade of TNF-*α* with IFX induced neutrophil apoptosis in patients with active CD but had a slight impact on patients with UC and healthy controls. One explanation for these results may be attributed to the discrepancy role of neutrophils playing in UC and CD. Inadequate clearance of invasive bacteria is responsible for prolonged inflammation in CD patients, while hyperreactivity is associated with persistent inflammation in UC patients. Although it has been reported that the induction of remission in IBD patients by IFX is mainly dependent on rapid blockade of neutrophil recruitment, we hypothesized that IFX-induced apoptosis of neutrophils may participate in the long-term remission. Therefore, our results reveal that delayed neutrophil apoptosis in active CD can be reversed by IFX treatment, which contributes to promoting intestinal mucosal healing.

## 5. Conclusions

This study establishes that production of proinflammatory mediators and migration of neutrophils are remarkably suppressed by anti-TNF-*α* therapy, which contributes to the induction of remission in active IBD. We found that the impact of IFX on neutrophils is mainly dependent on the restriction of MPO, ROS, calprotectin, and proinflammatory cytokine/chemokine production, inhibition of migration to inflamed intestinal mucosa, and induction of neutrophil apoptosis. These findings indicate that blockade of TNF-*α* modulates immune response of neutrophils in intestinal mucosa of patients with IBD and maintains intestinal homeostasis.

## Figures and Tables

**Table 1:** Clinical characteristics of IBD patients.

	Blood			Intestinal biopsy		
	HC	CD (A/R)^b^	UC (A/R)^b^	HC	CD (A/R)^b^	UC (A/R)^b^
Patients	24	26/22	28/16	10	15/7	12/0
Age (yrs)	52.5 ± 18	25.6 ± 6.8	41 ± 10	48.7 ± 17.4	24.7 ± 5.6	41.6 ± 11.7
Gender (F/M)	15/9	28/20	26/16	4/6	12/10	4/6
Duration (months)		28 ± 26.7	63.6 ± 46.2		27.5 ± 32	46.8 ± 28.5
Current therapy						
5-ASA		39	40		22	10
Immunosuppressants		27	14		5	4
Biologics		0	0		0	0
Disease extent^a^						
E1			5			
E2			22			3
E3			17			9
Disease location^a^						
L1		4			0	
L2		16			5	
L3		28			17	
L4		0			0	

^a^Montreal classification; ^b^A/R, active/remission.

## Data Availability

All data generated or analyzed during this study are included in this article.

## Abbreviations

CD: Crohn's disease
CDAI: Crohn's disease activity index
IBD: Inflammatory bowel disease
IFN-*γ*: Interferon *γ*
IL: Interleukin
TNF-*α*: Tumor necrosis factor *α*
UC: Ulcerative colitis.

## Supplementary Materials

Supplementary Materials Supplementary Figure 1. The purity of isolated peripheral neutrophils was 92%–95%. The peripheral neutrophils were isolated with Ficoll gradient centrifugation. After abandoning the red blood cells, neutrophils were measured with flow cytometry. Supplementary Figure 2. The expression of IL-1*β* (a), IFN-*γ* (b), TGF-*β* (c), and IL-17A (d) was detected by qRT-PCR. The production of IL-1*β* (e) and IFN-*γ* (f) was detected by ELISA. ^∗^ *P* < 0.05 and ^∗∗∗^ *P* < 0.001 compared with medium alone. ^##^ *P* < 0.01 and ^###^ *P* < 0.001 compared with LPS stimulation.
