# Supplementary material for: Anti-TNF-α Therapy Suppresses Proinflammatory Activities of Mucosal Neutrophils in Inflammatory Bowel Disease
Source: Mediators Inflamm. 2018 Nov 22;2018:3021863. doi: 10.1155/2018/3021863 (PMC6282128; doi:10.1155/2018/3021863)
Supplement: Supplementary Materials — Supplementary Figure 1. The purity of isolated peripheral neutrophils was 92%–95%. The peripheral neutrophils were isolated with Ficoll gradient centrifugation. After abandoning the red blood cells, neutrophils were measured with flow cytometry. Supplementary Figure 2. The expression of IL-1β (a), IFN-γ (b), TGF-β (c), and IL-17A (d) was detected by qRT-PCR. The production of IL-1β (e) and IFN-γ (f) was detected by ELISA. ∗ P < 0.05 and ∗∗∗ P < 0.001 compared with medium alone. ## P < 0.01 and ### P < 0.001 compared with LPS stimulation. [file 3021863.f1.pptx]

## Slide 1
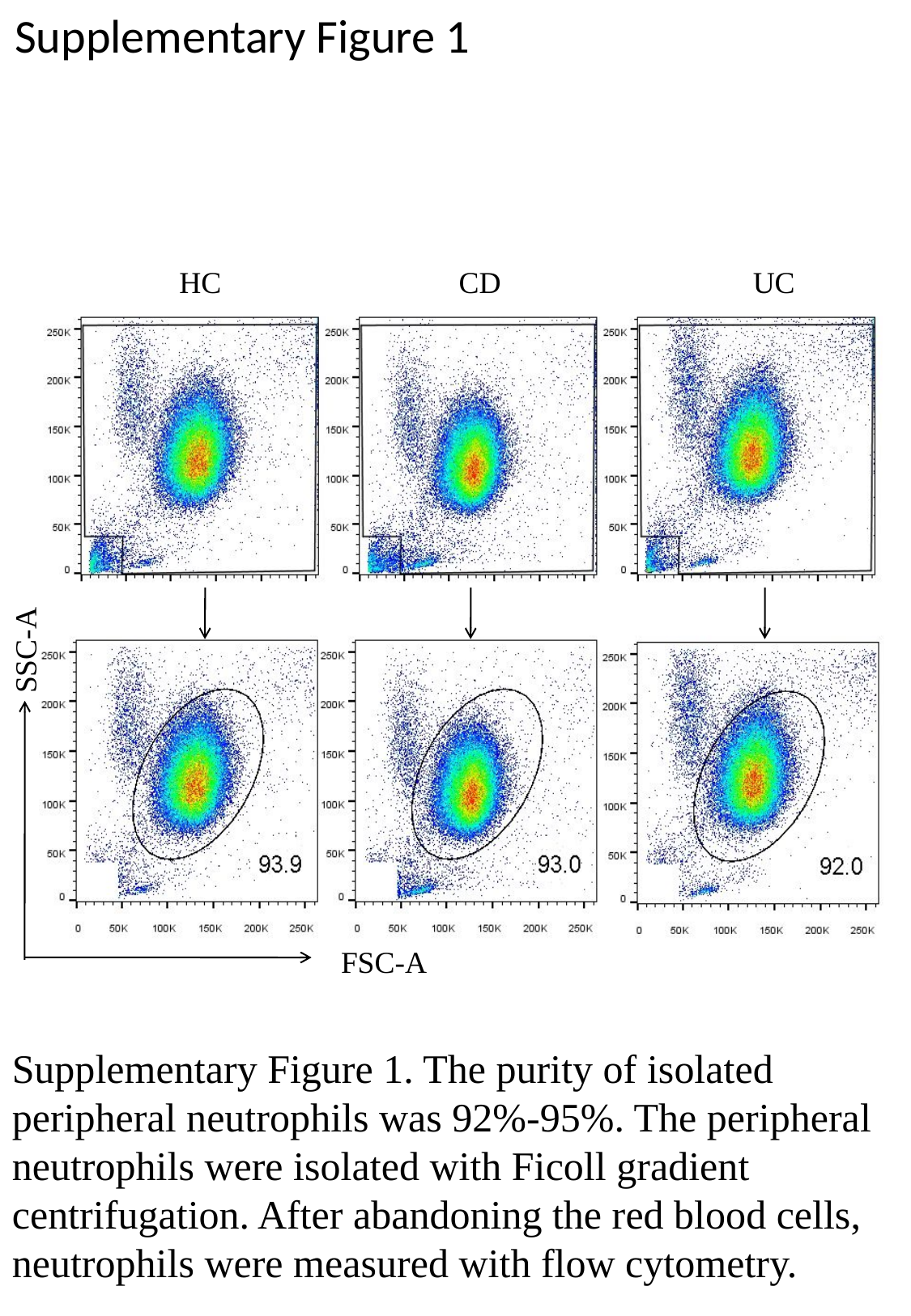

Supplementary Figure 1
HC
CD
UC
SSC-A
FSC-A
Supplementary Figure 1. The purity of isolated peripheral neutrophils was 92%-95%. The peripheral neutrophils were isolated with Ficoll gradient centrifugation. After abandoning the red blood cells, neutrophils were measured with flow cytometry.

## Slide 2
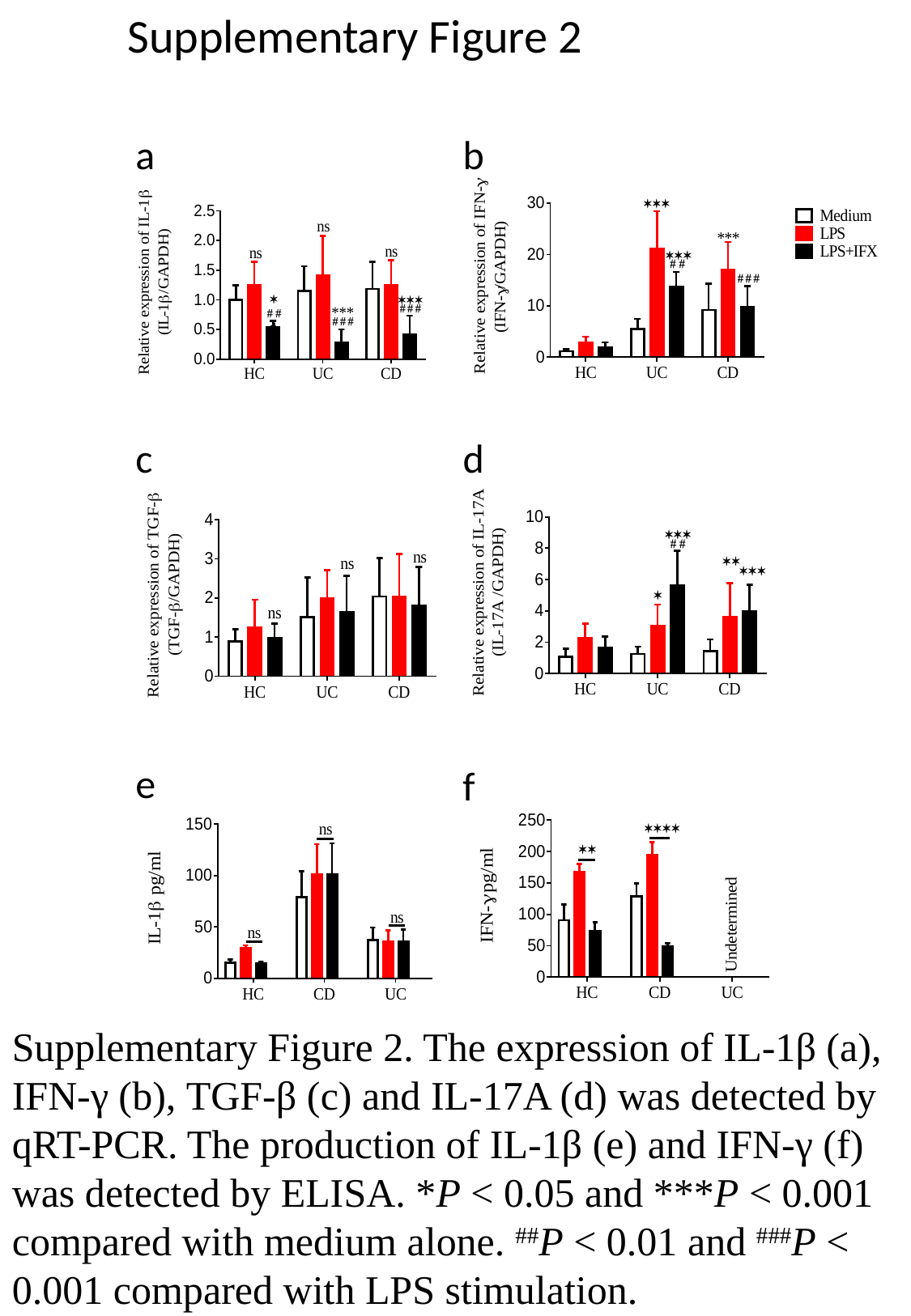

Supplementary Figure 2
a
c
e
b
d
f
Supplementary Figure 2. The expression of IL-1β (a), IFN-γ (b), TGF-β (c) and IL-17A (d) was detected by qRT-PCR. The production of IL-1β (e) and IFN-γ (f) was detected by ELISA. *P < 0.05 and ***P < 0.001 compared with medium alone. ##P < 0.01 and ###P < 0.001 compared with LPS stimulation.
